# miR-19b enhances osteogenic differentiation of mesenchymal stem cells and promotes fracture healing through the WWP1/Smurf2-mediated KLF5/β-catenin signaling pathway

**DOI:** 10.1038/s12276-021-00631-w

**Published:** 2021-05-25

**Authors:** Yan Huang, Yongqiang Xu, Siyin Feng, Pan He, Bing Sheng, Jiangdong Ni

**Affiliations:** 1 Department of Orthopaedics, Hunan Provincial People’s Hospital, Changsha, China; 2 Department of Orthopaedics, The Second Xiangya Hospital of Central South University, Changsha, China

**Keywords:** Diseases, Biotechnology

## Abstract

Bone marrow mesenchymal stem cell (BMSC)-derived exosomes have been found to enhance fracture healing. In addition, microRNAs contributing to the healing of various bone fractures have attracted widespread attention in recent years, but knowledge of the mechanisms by which they act is still very limited. In this study, we clarified the function of altered microRNA-19b (miR-19b) expression in BMSCs in fracture healing. We modulated miR-19b expression via mimics/inhibitors in BMSCs and via agomirs in mice to explore the effects of these changes on osteogenic factors, bone cell mineralization and the healing status of modeled fractures. Through gain- and loss-of function assays, the binding affinity between miR-19b and WWP1/Smurf2 was identified and characterized to explain the underlying mechanism involving the KLF5/β-catenin signaling pathway. miR-19b promoted the differentiation of human BMSCs into osteoblasts by targeting WWP1 and Smurf2. Overexpression of WWP1 or Smurf2 degraded the target protein KLF5 in BMSCs through ubiquitination to inhibit fracture healing. KLF5 knockdown delayed fracture healing by modulating the Wnt/β-catenin signaling pathway. Furthermore, miR-19b enhanced fracture healing via the KLF5/β-catenin signaling pathway by targeting WWP1 or Smurf2. Moreover, miR-19b was found to be enriched in BMSC-derived exosomes, and treatment with exosomes promoted fracture healing in vivo. Collectively, these results indicate that mesenchymal stem cell-derived exosomal miR-19b represses the expression of WWP1 or Smurf2 and elevates KLF5 expression through the Wnt/β-catenin signaling pathway, thereby facilitating fracture healing.

## Bone healing: Small RNA molecule implicated in fracture repair

Understanding how a small regulatory RNA molecule helps to promote fracture healing could lead to new treatments for broken bones. Working with human cells and mouse models, a team led by Yongqiang Xu from the Hunan Provincial People’s Hospital in Changsha, China, showed how microRNA-19b in extracellular vesicles secreted by bone marrow stem cells (BMSCs) contributes to the healing process. The researchers found that the microRNA blocks the function of two proteins that normally restrain the activity of a third protein needed for BMSCs to home in on the site of injury and turn into new bone tissue. In mice with leg bone fractures, injections of microRNA-19b–filled vesicles derived from BMSCs accelerated healing and recovery, suggesting that similar therapies might be helpful in human patients.

## Introduction

Bone fractures are the most frequently occurring type of large-organ, traumatic damage in humans^1^. The delayed healing process of bone fractures is a serious clinical and economic problem for both patients and health services^2^. The healing process of bone fractures is widely accepted as a complicated physiological course of events driven by early inflammatory reactions and is accompanied by various biological activities, such as osteogenic differentiation and biomineralization^3^. Fracture sites are commonly subjected to increasing oxidative stress after injuries, which impairs osteoblast function and impedes the process of fracture healing and remodeling^4^. The repair of fracture wounds is critically influenced by mechanical loading as well as the geometric configuration of the fractured fragments^5^. The recent available evidence on the molecular mechanism indicates that bone fracture healing involves the complex coordination of various cell types, proteins and genes to restore structural integrity^6^.

A limited amount of data have documented the paracrine mechanisms associated with the orchestration of mesenchymal stem cell (MSC) transplantation in bone fractures, and exosomes are pivotal elements of the paracrine effects^7^. MSC-derived exosome-mediated delivery of microRNAs (miRNAs), such as miR-126, has shown potential in accelerating fracture healing^8^. Of interest, upregulation of miR-19b could cause a significant increase in the transcription and translation of osteogenic factor genes during the process of osteogenic differentiation^9^. We used a bioinformatic prediction program to predict the potential target genes of miR-19b, and WWP1 and Smurf2 were predicted as the candidate target genes of miR-19b. Notably, the PY motif-containing transcription factor Kruppel like factor 5 (KLF5) is frequently degraded through ubiquitination by E3 ubiquitin ligases such as WWP1^10^. In addition, prior evidence has suggested that SMAD ubiquitination regulatory factor 2 (Smurf2), an E3 ubiquitin ligase, is an interacting protein of KLF5 and diminishes the protein stability of KLF5^11^. Furthermore, KLF5 has been revealed to activate β-catenin signaling in breast cancer^12^. In turn, Wnt/β-catenin signaling pathway activation has been proposed to enhance the production of osteogenic factors and thus expedite tibial fracture healing^13^. In this study, we sought to explain the regulatory mechanism of exosomal miR-19b derived from bone marrow mesenchymal stem cells (BMSCs) in the process of fracture healing, which may involve the WWP1/Smurf2/KLF5/β-catenin axis.

## Materials and methods

### Sample collection

The bone marrow of healthy volunteers at The Second Xiangya Hospital of Central South University was collected, and informed consent was obtained from the participants. This study was approved by the Ethics Committee of The Second Xiangya Hospital of Central South University. After surgery, portions of the fresh tissue samples were stored in liquid nitrogen within 30 min and were then transferred to a −80 °C freezer for further use.

### Isolation and culture of human BMSCs

Human BMSCs were isolated from healthy volunteers and amplified according to previously reported methods^14^. BMSCs below passage 4 were used in the following experiments. Maintenance medium comprising dulbecco’s modified Eagle’s medium (DMEM) (Invitrogen), 10% FBS and 100 U/ml penicillin/streptomycin (Invitrogen) was employed to incubate BMSCs at 37 °C in 5% CO_2_. The osteogenic differentiation induction medium was composed of 10% FBS, 50 mg/ml l-ascorbic acid, 10 mM glycerophosphate, and 100 nM dexamethasone and antibiotics (complete alpha-MEM) (Sigma, St. Louis, MO). Detection was performed on the 7th day after osteogenic differentiation was induced^15^.

### Extraction and identification of exosomes from BMSCs

Alpha-MEM (Gibco, Green Island) containing 20% FBS (Gibco) was centrifuged for 18 h at 200,000 *g* to deplete exosomes. BMSCs were cultured in exosome-free medium containing 10% FBS, and BMSCs at passages 4-6 were selected for exosome collection. A total of 2 × 10^6^ MSCs were cultured in 100 mm culture dishes under normal/hypoxic conditions for 72 h, and 10 ml of the supernatant was taken. Exosomes were then isolated from the supernatant after centrifugation^16^. Then, exosomes were resuspended in PBS.

For identification of exosomes, samples were evaluated under a JEM-2100 transmission electron microscope (TEM; JEOL, Tokyo, Japan). Images were acquired using the PARTICLEMEIRIX system. Nanoparticle tracking analysis (NTA) was performed using the NanoSight NS300 system (Malvern Instruments, Malvern, UK). The Brownian motion of exosomes in PBS was recorded and tracked, and the size distribution was analyzed using the Stokes-Einstein equation. The exosome characteristics were identified by detecting the expression of the exosome-specific surface markers with rabbit anti-CD63 (1:2000, ab216130, Abcam, UK), rabbit anti-TSG101 (1:10,000, ab125011, Abcam), rabbit anti-CD81 (1:10,000, ab109201, Abcam) and rabbit anti-Calnexin (1:100,000, ab92573, Abcam) antibodies by Western blot analysis.

### Experimental protocols

When the cell density was 90% and the cells were in in logarithmic growth phase, trypsin digestion and pipetting were performed to prepare a 2.5 × 10^4^ cell/ml cell suspension, which was added to a 6-well plate at 2 ml per well. When the cells were in logarithmic growth phase and were 30% confluent, the corresponding lentiviruses (2 × 10^6^ TU) and 5 μg of polybrene were added to 1 ml of serum/antibacterial drug-free medium. Forty-eight hours after transduction, 1 μg/ml puromycin was added to each well to select stably transduced cells. After successful lentiviral infection, cells at a confluence of 70% to 80% were subjected to transient transduction of the miR-19b mimic/inhibitor, WWP1 overexpression plasmid (oe-WWP1), Smurf2 overexpression plasmid (oe-Smurf2), KLF5 overexpression plasmid (oe-KLF5), β-catenin overexpression plasmid (oe-β-catenin), shRNA targeting KLF5 (sh-KLF5) or the corresponding negative controls (NCs) according to the instructions for Lipofectamine^TM^ 2000 (Invitrogen). Cells were collected 48 h after transduction, and the transduction efficiency was determined for subsequent experiments.

### Western blot analysis

The transduced cells and fracture healing tissues harvested at 2 and 4 weeks after fracture were lysed with RIPA lysis and extraction buffer (Thermo Fisher Scientific). Protease inhibitors and phosphatase inhibitors were added for lysis for 30 min. The protein concentration was determined using a Bio-Rad protein assay kit (Bio-Rad). Extracts were resolved by SDS-PAGE and electroblotted onto nitrocellulose membranes (Bio-Rad). The membranes were probed with primary antibodies against rabbit WWP1 (ab104440), Smurf2 (ab94483), KLF5 (ab137676), β-catenin (ab32572), Runx2 (ab23981), Collagen I (ab34710), ALP (ab83259), and β-actin (1:1000, ab5694) overnight at 4 °C. Next, the membranes were incubated with HRP-conjugated goat anti-rabbit IgG secondary antibodies (ab6721). The antibodies were purchased from Abcam, Cambridge, UK. The blots were developed with ECL reagent (BB-3501, Amersham, UK). Images were acquired with a Bio-Rad image analysis system (BIO-RAD) and quantified with Quantity One v4.6.2 software. Total cell protein and cytoplasmic protein levels were internally normalized to β-actin levels to determine relative protein levels.

### Ubiquitination assay

For the ubiquitination assay, BMSCs were treated with the proteasome inhibitor MG132 (20 μM, ab141003, Abcam) for 4 h. Via incubation of the whole-cell lysate (500 μg protein/sample) with UbiCapture-Q Matrix (Biomol) overnight under gentle stirring at 4 °C, all ubiquitinated proteins were pulled down. After three washes, the captured proteins were eluted with 2× SDS-PAGE loading buffer. Cycloheximide was added according to the kit’s instruction manual (Sigma, St. Louis, Mo) at a concentration of 100 ng/ml. Western blot analyses were adopted to examine the total KLF5 protein level at 0, 6, 12, and 24 h.

### RNA immunoprecipitation (IP)-Western blot analysis

Coimmunoprecipitation was performed using anti-myc and anti-FLAG antibodies, as well as an anti-FLAG monoclonal antibody (F1804, Sigma), anti-myc monoclonal antibody (M5546, Sigma), and IgG goat anti-rabbit polyclonal antibody (ab20272, 1:5000, Abcam, UK). Then, transduced BMSCs were lysed on ice for 5 min. Then, the cell lysate was sonicated 4 times on ice (5 s each) and centrifuged at 4 °C for 10 min to harvest the supernatant (200 µl), which was incubated with the primary anti-myc antibody (2 µl) at 4 °C overnight. Next, the solution was incubated with 50% Protein A-agarose was at 4 °C for 2 h. The beads were washed five times with 500 µl of 1 × cell lysate. The protein was suspended in 50 µl of SDS sample buffer and analyzed by Western blotting.

### GST pull-down assay

The GST fusion protein was purified from bacterial DNA fragments. WWP1 and Smurf2 were amplified and cloned into the pGEX-6p-1 GST fusion protein vector (Amersham Biosciences). The purified GST fusion protein was eluted into 10 mm reduced glutathione. The protein concentration was determined by the Bradford method.

The wild-type KLF5 and its mutant DNA template translated in vitro were used by PCR using the forward primer: 5′-GGATCCTAATACGACTCACTATAGGAACAGACCACC**ATG**GCTACAAGGGTGCTGAG-3′ (the T7 promoter sequence is underlined; the start codon ATG is in bold font) and reverse primer: 5′-TTT**CTA**GACTACTTGTCATCGT CGTCCTTGTAATCGTTCTGGTGCCTCTTCATAT-3′ (the stop codon is in bold font). RNA transcription and protein translation were carried out in vitro with Quick Coupled Transcription/Translation Systems (Promega) using [^35^S]-methionine (Amersham Biosciences).

The purified GST fusion proteins (GST, GST-WWP1, or GST-Smurf2) were pulled down in GST binding buffer and conjugated to 50 µl of 50% glutathione-Sepharose 4B suspension beads. After incubation at 4 °C for 1 h, the cells were incubated with GST pulldown binding buffer. Then, 10 µl of ^35^S-labeled in vitro translated protein (KLF5) was added and incubated at 4 °C for 2 h with shaking. Bound proteins were eluted. GST-tagged proteins were visualized with Coomassie blue staining, and ^35^S-labeled proteins were assessed by autoradiography.

### In vivo protein ubiquitination conjugation assay

The in vitro ubiquitination assay was carried out by using a ubiquitin-protein conjugation kit (Boston Biochem, Cambridge, MA). A rabbit anti-reticulocyte lysate-translated, ^35^S-labeled KLF5 antibody was used to observe the existence of GST-WWP1 or GST-Smurf2. The samples were subjected to 10% SDS-PAGE for autoradiography.

### Alizarin red staining (ARS) and alkaline phosphatase (ALP) staining

After osteogenic differentiation induction for 2 weeks, cells were washed twice with PBS and fixed with 4% paraformaldehyde. Cells were stained with 2% ARS (Sigma, pH 4.2) to visualize matrix calcium deposition and were then subjected to ALP staining using nitroblue tetrazolium (NBT)/5-bromo-4-chloro-3-indolyl-phosphate (Sigma).

### Luciferase activity assay

The full-length WWP1 3′UTR sequence (WT) and Smurf2 3’UTR sequence (WT) were obtained from the NCBI database and inserted downstream of the firefly luciferase gene driven by the SV40 promoter in the pEZX-MT01 vector to generate the recombinant vectors PEZX-MT01-3′UTR WWP1-Mut and PEZX-MT01-3′UTR Smurf2-Mut. PEZX-MT01-3′UTR WWP1-Wt or PEZX-MT01-3′UTR WWP1-Mut was cotransfected with the miR-19b mimic NC, miR-19b mimic, anti-miR-19b mimic NC or anti-miR-19b into 293 T cells. Cells were cultured for 48 h using Lipofectamine 2000 (Life Technologies). Luciferase activity was quantified using an EnVision 2102 Multilabel Plate Reader (Perkin Elmer Inc., Waltham, MA), with Renilla luciferase as the internal reference.

### Fracture model establishment and experimental grouping

All animal experiments were performed in accordance with the principles and procedures approved by the Laboratory Animal Ethics Committee of The Second Xiangya Hospital of Central South University. Transverse femoral shaft fractures were generated in C57 male mice (8 weeks old) using a C-shaped instrument with a three-point bend. The right knee was exposed by using the lateral metatarsal approach, and a medial metatarsal dislocation was generated. The femoral intercondylar sulcus was exposed at the knee joint by complete flexion, and a 0.5 mm diameter drill hole was made in the center of the intercondylar sulcus. After minimal lateral exposure, a saw was used to cut a thin (3 mm deep) line along the central axis to weaken the bones. The right femur of each animal was fractured by a three-point bending technique^17,18^. The muscle fascia and skin were closed. After successful model establishment, the mice were randomly divided into 8 groups with 10 mice in each group. Except for those in the control group (sham operation), all mice were injected with lentivirus-based transduction agents (25 mg/100 ml) carrying the miR-19b agomir, oe-WWP1, oe-Smurf2, sh-KLF5 or sh-β-catenin alone or in combination at the fracture site after fracture. Photographing and identification of harvested tissue samples were performed at different time points.

### Injection of exosomes and grouping

After establishment of the fracture model using the abovementioned methods, the successfully established model mice were randomly divided into three groups with 10 mice in each group to explore whether BMSC-derived exosomes accelerate fracture healing in vivo. After isolation and identification of exosomes from MSCs, 200 µg of exosomes was injected around the fracture area, and the mice were grouped into the control, PBS, and exo groups.

### X-ray examination for evaluation of the wound area

Radiographic imaging was performed at 0 weeks (the day of fracture) and 4 weeks after fracture. A digital X-ray imaging system (30 kV, 8 mAs) was adopted to for X-ray imaging of the right femur fractures in the mice. Mice were observed for fracture healing and callus growth, and the wound area was then measured.

### Hematoxylin-eosin (HE)/Alcian blue staining for evaluation of mineralized callus area

Femora were collected at the 4th week after fracture. These samples were fixed in 4% paraformaldehyde at 4 °C for 24 h and embedded in paraffin. The bone specimens were fixed at room temperature and decalcified for 2 weeks. The decalcified bone was then dehydrated and embedded in paraffin using the Leica EG embedding system (Leica Microsystems, Wetzlar, Germany). The sections were stained with hematoxylin, eosin and Alcian blue (Sigma). Histomorphological analysis was performed using Zen2012 (Zeiss, Oberkochen, Germany), with evaluation of the mineralized area.

### Quantification of gene expression

Total RNA was extracted using an RNeasy Mini Kit (Qiagen, Valencia, CA), and mRNA was reverse transcribed to cDNA using a reverse transcription kit (RR047A, Takara, Japan). For miRNA detection, a miRNA First Strand cDNA Synthesis (Tailing Reaction) kit (B532451-0020, Sangon, Shanghai, China) was used for reverse transcription to obtain cDNA. Samples were loaded using a SYBR® Premix Ex TaqTM II (Perfect Real Time) kit (DRR081, Takara, Japan) and were analyzed by qRT-PCR in a real-time quantitative PCR instrument (ABI 7500, ABI, Foster City, CA). Three replicate wells were established per sample. The miRNA universal negative primer and the U6 internal reference upstream primer were provided in the miRNA First Strand cDNA Synthesis (Tailing Reaction) kit. The other primer sequences for GAPDH and U6 as housekeeping genes are shown in Table 1 and were designed and provided by Sangon Biotech (Shanghai, China). The relative expression levels of the products were calculated using the formula 2^-ΔΔCt^.

**Table 1:** Primer sequences for qRT-PCR.

Gene	Human	Mouse
WWP1	F: 5′-GTATGGATCCTGTACGGCAGCA-3′	F: 5′-ACCCAAGAGCCTCCTGTA-3′
	R: 5′-GTTGTGGTCTCTCCCATGTGGT-3′	R: 5′-CTCTGTTCCCACCCTGAT-3′
Smurf2	F: 5′-GGCTCAATTCTTGGCTCTG-3′	F: 5′-CAGCACCTGCTGAAGACATT-3′
	R: 5′-ACCGGGTGTTTACCTTCC-3′	R: 5′-GAACCACTTGACGACAT TGC-3′
KLF5	F: 5′-TCCACGCCACTAAGCATGTG-′	F: 5′-ACTGCCCTCGGAGGAGCTGG-3′
	R: 5′-CGTAAATGACCGTCCTGGTCTT-3′	R: 5′-ATGCTCTGAAATTATCGGAACTG-3′
β-catenin	F: 5′-AGTTGAGCACCTGTTTGCCTGA-3′	F: 5′-GCATCAATGGCTCAAGGACAAG-3′
	R: 5′-ATGAGCAGCACTCGGACCTTC-3′	R: 5′-CCGGTCTCCAATTCCCAAGATA-3′
hsa-miR-19b	5′-GCAAATCCATGCAAAACTGA-3′	

*F* forward, *R* reverse.

### Statistical analysis

The data are expressed as the mean ± standard deviation values calculated by SPSS 21.0 statistical software (IBM Corp, Armonk, NY). For comparisons between two groups, an independent samples *t*-test was conducted. For multiple comparisons, one-way analysis of variance (ANOVA) followed by Tukey’s post hoc test was performed. Differences were considered statistically significant when *p* < 0.05.

## Results

### miR-19b promotes the differentiation of human BMSCs into osteoblasts by targeting WWP1 and Smurf2

**Fig. 1:**
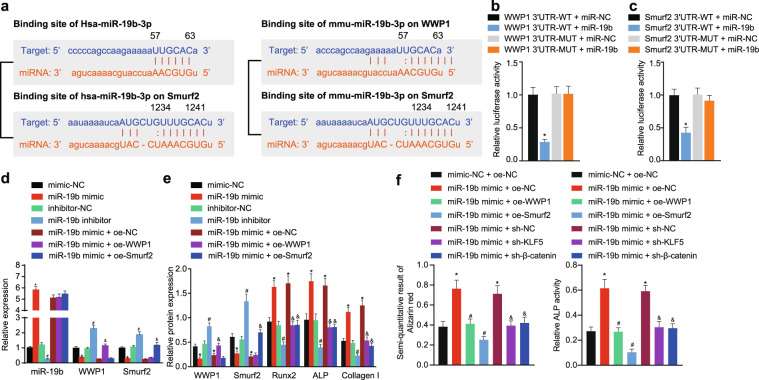
miR-19b promotes the differentiation of human BMSCs into osteoblasts by targeting WWP1 and Smurf2. **a** Bioinformatic analysis (TargetScan) predicted that the 3′UTRs of WWP1 and Smurf2 are complementary to the miR-19b seed region. **b** The wild-type WWP1 3′UTR (named WWP1 3’UTR-WT) and mutant WWP1 3′UTR (named WWP1 3′UTR-MUT; UUGCAC was mutated to CAUUGG) were constructed with the miR-19b binding sites, and luciferase activity was measured with a dual luciferase reporter assay. **c** The wild-type Smurf2 3′UTR (named Smurf2 3′UTR-WT) and mutant Smurf2 3′UTR (named Smurf2 3′UTR-MUT; UUUGCA was mutated to ACGUUU) were constructed with the miR-19b binding sites, and luciferase activity was measured with a dual luciferase reporter assay. **d** The expression level of miR-19b and mRNA expression of WWP1 and Smurf2 in human BMSCs was detected by qRT-PCR, *p < 0.05 versus the mimic-NC group, ^#^*p* < 0.05 versus the inhibitor-NC group, ^&^*p* < 0.05 versus the miR-19b mimic + oe-NC group. **e** The protein levels of WWP1, Smurf2, Runx2, ALP, and Collagen I in human BMSCs were determined by Western blot analysis. **f** Quantitative analysis of Alizarin red staining and ALP staining was performed to determine the degree of mineralization in human BMSCs. The experimental results are measurement data and are expressed as the mean ± standard deviation values. For comparisons between two groups, an independent samples *t* test was conducted. For multiple comparisons, one-way ANOVA followed by Tukey’s post hoc test was performed. The experiment was repeated 3 times.

Bioinformatic analysis predicted that miR-19b targets WWP1 and Smurf2 and indicated that the 3′UTRs of WWP1 and Smurf2 are complementary to the miR-19b seed region (Fig. 1a). This targeting effect exists in both humans and mice.

The luciferase reporter assay results (Fig. 1b, c) illustrated that miR-19b bound to the 3′UTR of WT WWP1 and Smurf2. Relative to that in the WWP1 3′UTR-WT + miR-NC group, the luciferase activity in the WWP1 3′UTR-WT + miR-19b mimic group was appreciably reduced (*p* < 0.05), while there was no significant difference in the corresponding two Mut groups (*p* > 0.05). Relative to that in the Smurf2 3′UTR-WT + miR-NC group, the luciferase activity in the Smurf2 3′UTR-WT + miR-19b mimic group was appreciably reduced (*p* < 0.05), and no significant difference was observed in luciferase activity in the corresponding two Mut groups (*p* > 0.05).

Subsequent experiments were performed using human BMSCs. In response to miR-19b mimic treatment, the mRNA levels of WWP1 and Smurf2 were appreciably reduced but were elevated in the presence of the miR-19b inhibitor. In addition, the mRNA level of WWP1 or Smurf2 was increased in response to combined treatment with miR-19b mimic + oe-WWP1 or miR-19b mimic + oe-Smurf2, respectively, but the influence of the miR-19b mimic on the WWP1 or Smurf2 mRNA level was abolished in the presence of the Smurf2 or WWP1 overexpression plasmid, respectively (Fig. 1d). Consistent results were observed in the Western blot analysis; miR-19b mimic treatment resulted in decreased protein levels of WWP1 and Smurf2, and treatment with the miR-19b inhibitor increased these levels. Furthermore, the miR-19b mimic led to increases in the protein levels of Runx2, ALP, and Collagen I, and the miR-19b inhibitor caused reductions in these levels (Fig. 1e). The ARS staining and ALP staining results (Fig. 1f) revealed a reduced proportion of mineralization in response to the miR-19b inhibitor; in contrast, mineralization was promoted by the miR-19b mimic. Treatment with oe-WWP1 and oe-Smurf2 suppressed the miR-19b mimic-induced increase in the proportion of mineralization. These data indicate that miR-19b targets and inhibits WWP1 and Smurf2 expression to enhance the differentiation of human BMSCs into osteoblasts.

### WWP1 or Smurf2 degrades KLF5 by ubiquitination to repress fracture healing

**Fig. 2:**
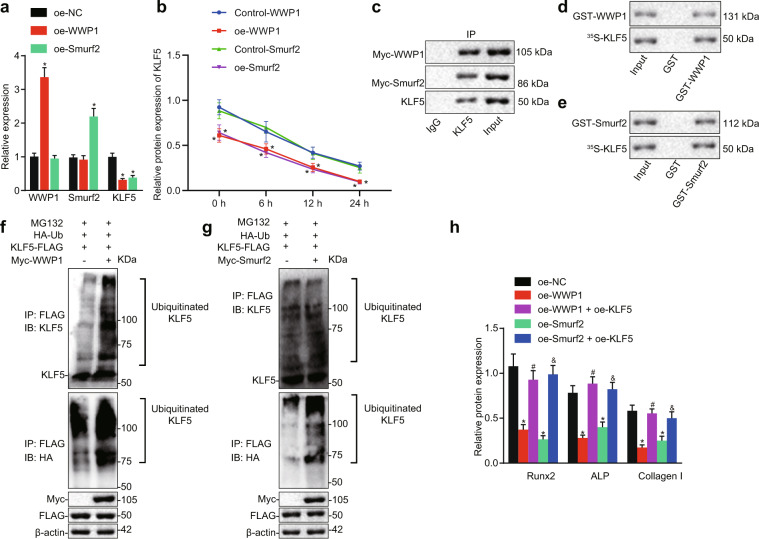
Overexpression of WWP1 or Smurf2 degrades their target protein KLF5 in BMSCs through ubiquitination to inhibit fracture healing. **a** PCR was adopted to examine the mRNA levels of WWP1, Smurf2, and KLF5. *p < 0.05 versus the oe-NC group. **b** After cycloheximide treatment (100 ng/ml), the total protein level of KLF5 was measured by Western blot analysis at 0, 6, 12, and 24 h. **c** Myc was used to label WWP1 and Smurf2, and endogenous KLF5 was detected with input as the positive control and IgG as the negative control. **d**–**e** GST pulldown assays were performed using GST-WWP1 (**d**) or GST-Smurf2 (**e**) fusion proteins and in vitro-translated KLF5 protein to examine exogenous KLF5 expression. Input is the positive control, and GST is the negative control. **f**–**g** Ubiquitin was added to examine ubiquitination of KLF5 after overexpression of WWP1 (**f**) or Smurf2 (**g**). **h** Western blot analysis of the protein levels of Runx2, ALP, and Collagen I with β-actin as the internal reference protein. The relative protein expression levels of Runx2, ALP, and Collagen I. *p < 0.05 versus the oe-NC group, ^#^*p* < 0.05 versus the oe-WWP1 + oe-KLF5 group, ^&^*p* < 0.05 versus the oe-Smurf2 + oe-KLF5 group. The experimental results are measurement data and are expressed as the mean ± standard deviation values. For multiple comparisons, one-way ANOVA followed by Tukey’s post hoc test was performed. The experiment was repeated 3 times.

Next, we sought to explain the mechanism of the E3 ligases WWP1 and Smurf2 on the ubiquitination of KLF5 in BMSCs. The transduction efficiency in BMSCs was evaluated after transduction of the WWP1 and Smurf2 overexpression plasmids. The PCR results validated the good transduction efficiency of oe-WWP1 and oe-Smurf2. In addition, treatment with oe-WWP1 and oe-Smurf2 resulted in reduced KLF5 expression (Fig. 2a). Western blot analyses were performed at 0, 6, 12, and 24 h to examine total KLF5 protein levels, as shown in Fig. 2b. After overexpression of WWP1 or Smurf2, the degradation rate of the KLF5 protein in BMSCs increased with time.

IP assays and GST pulldown assays were carried out to determine whether WWP1 or Smurf2 directly interacts with KLF5 at the protein level. Myc-tagged WWP1 and Smurf2 were expressed in cells to detect endogenous KLF5. As shown in Fig. 2c, myc-WWP1 and myc-Smurf2 were immunoprecipitated by the anti-KLF5 antibody but not by IgG as the control. These results indicate that WWP1 and Smurf2 interact with endogenous KLF5 in BMSCs. A GST pulldown assay was carried out using the GST-WWP1 and GST-Smurf2 fusion proteins and the in vitro-translated KLF5 protein. The wild-type proteins (GST-WWP1 and GST-Smurf2) pulled down the KLF5 protein, but the GST protein alone could not bind to the KLF5 protein under the same conditions (Fig. 2d, e).

The ubiquitination assay was performed under the IP experimental conditions with FLAG, and the effect on KLF5 ubiquitination after overexpression of WWP1 or Smurf2 was assessed by adding ubiquitin (Fig. 2f, g). WWP1 and Smurf2 were adopted to induce ubiquitination of endogenous KLF5 protein in cells. After treatment with MG132 (20 μM, 4 h), ubiquitination of KLF5 by WWP1 or Smurf2 was observed during incubation with myc-tagged WWP1 or Smurf2, respectively, via Western blot analysis with an anti-KLF5 antibody. When Western blot analysis was performed using an anti-HA tag antibody for labeling, the ubiquitination of KLF5 by myc-tagged WWP1 or Smurf2 was more obvious. These results provide direct evidence that WWP1 and Smurf2 are the E3 ubiquitin ligases of KLF5.

After co-transduction of oe-WWP1 or oe-Smurf2 and oe-KLF5, the changes in the Runx2, ALP, and Collagen I protein levels were assessed by Western blot analysis (Fig. 2h). Treatment with oe-WWP1 or oe-Smurf2 alone diminished the protein levels of Runx2, ALP and Collagen I. However, combined treatment with oe-KLF5 and oe-WWP1 or oe-Smurf2 led to higher protein levels of Runx2, ALP and Collagen I than those generated in response to treatment with oe-WWP1 or oe-Smurf2 alone. The data suggested that WWP1 and Smurf2 degraded their target protein KLF5 through ubiquitination to inhibit fracture healing.

### KLF5 knockdown delays fracture healing by modulating the Wnt/β-catenin signaling pathway

**Fig. 3:**
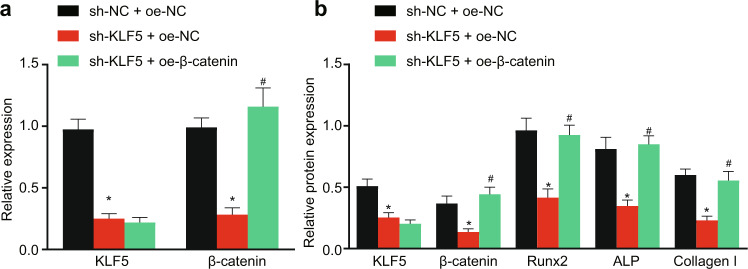
KLF5 knockdown delays fracture healing by modulating the Wnt/β-catenin signaling pathway. Primary human BMSCs were transduced with lentiviral vectors, and osteogenic differentiation was induced for 7 days. **a** The mRNA levels of KLF5 and β-catenin were measured by qPCR. *p < 0.05 versus the sh-NC + oe-NC group, ^#^*p* < 0.05 versus the sh-KLF5 + oe-NC group. **b** Western blot analysis of the protein levels of KLF5, β-catenin, Runx2, ALP, and Collagen I, with β-actin as the internal reference protein. *p < 0.05 versus the sh-NC + oe-NC group, ^#^*p* < 0.05 versus the sh-KLF5 + oe-NC group. The experimental results are measurement data and are expressed as the mean ± standard deviation values. For multiple comparisons, one-way ANOVA followed by Tukey’s post hoc test was performed. The experiment was repeated 3 times.

Evidence indicates that KLF5 is involved in the regulation of β-catenin in breast cancer^12^. Additionally, it has been reported that the Wnt/β-catenin signaling pathway is involved in fracture repair^13^. Furthermore, we clarified whether KLF5 plays a role in modulating the Wnt/β-catenin signaling pathway during fracture healing. We downregulated KLF5 and measured the mRNA levels of the Wnt/β-catenin signaling pathway-related factor β-catenin in cells by qPCR. As shown in Fig. 3a, sh-KLF5 treatment reduced the mRNA levels of KLF5 and β-catenin, and oe-β-catenin appreciably increased the level of β-catenin that was decreased by sh-KLF5. In addition, consistent results were revealed by Western blot analysis, which verified that oe-β-catenin markedly elevated the protein level of β-catenin that was decreased by sh-KLF5. Furthermore, the decreases in the protein levels of Runx2, ALP, and Collagen I induced by sh-KLF5 were rescued by oe-β-catenin (Fig. 3b). These data indicate that KLF5 knockdown delays fracture healing by modulating the Wnt/β-catenin signaling pathway.

### miR-19b enhances fracture healing via the KLF5/β-catenin signaling pathway by targeting WWP1 or Smurf2

**Fig. 4:**
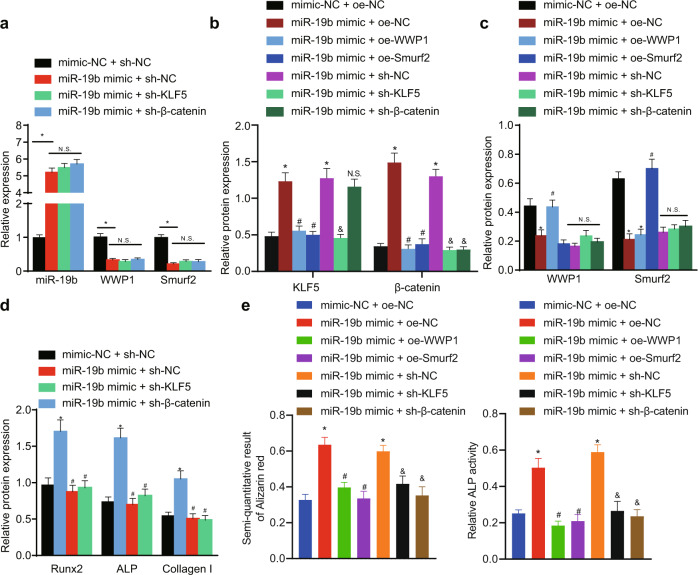
miR-19b enhances fracture healing *via* the KLF5/β-catenin signaling pathway by targeting WWP1 or Smurf2. Primary human BMSCs were transduced with lentiviral vectors followed by induction of osteogenic differentiation for 7 days. **a** The expression level of miR-19b and mRNA levels of WWP1 and Smurf2 were measured by qPCR. *p < 0.05 versus the mimic-NC + sh-NC group, NS *p* > 0.05 versus the miR-19b mimic + sh-NC group. **b**–**c** Western blot analysis of the protein levels of KLF5 (**b**), β-catenin (**b**), WWP1 (**c**), and Smurf2 (**d**), with β-actin as the internal reference protein. *p < 0.05 versus the mimic-NC + sh-NC group, ^#^*p* < 0.05 versus the miR-19b mimic + oe-NC, ^&^*p* < 0.05 versus the miR-19b mimic + sh-NC group, NS *p* > 0.05. **d** Western blot analysis of the protein levels of Runx2, ALP, and Collagen I, with β-actin as the internal reference protein. *p < 0.05 versus the mimic-NC + sh-NC group, ^#^*p* < 0.05 versus the miR-19b mimic + sh-NC group. **e** Quantitative analysis of ARS staining and ALP staining was performed to determine the degree of cell mineralization. The experimental results are measurement data and are expressed as the mean ± standard deviation values. For multiple comparisons, one-way ANOVA followed by Tukey’s post hoc test was performed. The experiment was repeated 3 times.

Primary human BMSCs were co-transduced with the miR-19b mimic and sh-KLF5 or sh-β-catenin, and osteogenic differentiation was then induced for 7 days. The miR-19b mimic successfully induced miR-19b overexpression, which in turn resulted in reductions in the mRNA levels of WWP1 and Smurf2. However, combined treatment with miR-19b mimic + sh-KLF5/sh-β-catenin abolished the effects of the miR-19b mimic on the levels of miR-19b, WWP1 and Smurf2 (Fig. 4a).

Primary human BMSCs were transfected with the miR-19b mimic, oe-WWP1, oe-Smurf2, or sh-β-catenin alone or in combination. The results of qPCR after 7 days of osteogenic differentiation are shown in Supplementary Fig. S1a. It was shown that overexpressing miR-19b elevated the mRNA expression levels of KLF5 and β-catenin and that this miR-19b mimic-induced upregulation of expression was appreciably reduced by oe-WWP1, oe-Smurf2, or sh-KLF5. Combined treatment with the miR-19b mimic + sh-β-catenin diminished the β-catenin level but did not significantly alter the level of KLF5. Western blot analysis, as shown in Fig. 4b, c, verified that the miR-19b mimic decreased the levels of WWP1 and Smurf2, yet the addition of oe-WWP1 to the miR-19b mimic upregulated WWP1, and combined treatment with the miR-19b mimic and oe-Smurf2 upregulated Smurf2 in BMSCs.

Additionally, the miR-19b mimic elevated the levels of Runx2, ALP, and Collagen I, while this miR-19b mimic-induced upregulation was appreciably reduced by sh-KLF5 or sh-β-catenin (Fig. 4d). In addition, the ARS staining and ALP staining results (Fig. 4e) revealed that the proportion of mineralization was increased by the miR-19b mimic and that this increase was reversed by oe-WWP1, oe-Smurf2, or sh-KLF5. These data indicate that miR-19b can enhance fracture healing by targeting WWP1 or Smurf2 expression and modulating the KLF5/β-catenin signaling pathway.

### miR-19b enhances fracture healing in mice through the WWP1/Smurf2/KLF5/β-catenin axis

**Fig. 5:**
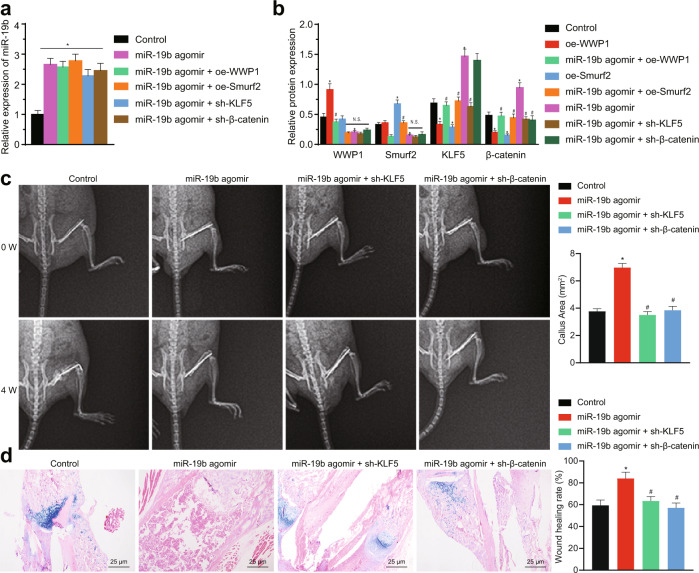
miR-19b promotes fracture healing in mice through the KLF5/β-catenin axis. Mice in the femoral fracture model were injected with transduction reagents after the operation. **a** On the 7th day after injection and in week 0 after fracture, calli were harvested from mice in each group, and the level of miR-19b was measured by PCR to confirm the transduction efficiency of the miR-19b agomir in mice. *p < 0.05 versus the control group. **b** In week 0 after fracture, calli were harvested from mice in each group, and the protein levels of WWP1, Smurf2, KLF5, and β-catenin were measured by Western blot analysis, with β-actin as the internal reference protein. *p < 0.05 versus the control group, ^#^*p* < 0.05 versus the miR-19b agomir group, NS > 0.05. **c** Representative X-ray images acquired to monitor femoral healing and changes over time at weeks 0 and 4 after fracture. **d** At the 4th week after femoral fracture, callus sections were stained with HE/Alcian blue, and the histology of the fracture calli was observed. Blue indicates cartilage, and pink indicates mineralized callus area. The experimental results are measurement data and are expressed as the mean ± standard deviation values. For multiple comparisons, one-way ANOVA followed by Tukey’s post hoc test was performed. *n* = 5 in the weeks 0 and 4 after fracture.

To further confirm the effect of miR-19b on fracture healing in vivo, a femoral fracture model was established in mice. X-ray and micro-computed tomography (μCT) imaging were adopted to assess the femoral fractures (Fig. 5a) (Due to the large number of groups, the data in the Fig. 5 only show the untreated group of the fracture model as the Control group; the other control groups are not shown). On the 7th day after injection, the calli were harvested from the mice for evaluation of miR-19b expression to confirm the transduction efficiency (Fig. 5a). In the mice, treatment with the miR-19b agomir resulted in diminished protein levels of WWP1 and Smurf2 and elevated protein levels of KLF5 and β-catenin. Reductions were observed in the protein levels of KLF5 and β-catenin in response to oe-WWP1 and oe-Smurf2. In addition, combined treatment with the miR-19b agomir + oe-WWP1 or oe-Smurf2 led to increased levels of WWP1 or Smurf2, respectively, and decreased levels of KLF5 and β-catenin but did not affect the levels of Smurf2 or WWP1. Combined treatment with miR-19b agomir + sh-KLF5 diminished the KLF5 and β-catenin levels but did not affect the WWP1 and Smurf2 levels. Combined treatment with the miR-19b agomir + sh-β-catenin diminished the β-catenin level but did not affect the level of KLF5, WWP1 or Smurf2 (Fig. 5b). Together, these results indicate that miR-19b enhances KLF5/β-catenin expression by inhibiting WWP1/Smurf2 in vivo.

At 0 and 4 weeks after the fracture model was established, the healing area of internal bone formation during the healing process of the femoral fractures in mice was evaluated by X-ray imaging (Fig. 5c; Supplementary Fig. S1b). Quantification of the healing area showed that relative to the control group at week 4, the miR-19b agomir group had a larger healing area; in addition, the remodeling process was observed, indicating that bone repair was accelerated. The healing area in the oe-WWP1 group and the oe-Smurf2 group at the 4th week were not appreciably different from the healing area in the control group, indicating that bone repair was slow. Moreover, there was no significant difference in fracture healing at the 4th week in the miR-19b agomir + oe-WWP1 group, the miR-19b agomir + oe-Smurf2 group, the miR-19b agomir + sh-KLF5 group, or the miR-19b agomir + sh-β-catenin group relative to the control group.

At the 4th week after fracture, fracture calli were decalcified for tissue sectioning and was then stained with HE/Alcian blue for quantification of wound healing (Fig. 5d; Supplementary Fig. S1c). At the 4th week, miR-19b agomir treatment had resulted in a higher mineralization rate, better fracture wound healing, and accelerated fracture healing. Treatment with oe-WWP1 and oe-Smurf2 caused lower mineralization rates, slower wound healing and a later onset of fracture healing. The results of combined treatment with miR-19b agomir + oe-WWP1, miR-19b agomir + oe-Smurf2, miR-19b agomir + sh-KLF5, and miR-19b agomir + sh-β-catenin were not appreciably different from those of the control treatment. Among these results, relative to the oe-WWP1 group, the miR-19b agomir + oe-WWP1 group had a higher mineralization rate and better fracture wound healing. Relative to the oe-Smurf2 group, the miR-19b agomir + oe-Smurf2 group had a higher mineralization rate and better fracture wound recovery. Relative to miR-19b agomir treatment, the miR-19b agomir + sh-KLF5 and miR-19b agomir + sh-β-catenin treatments resulted in a lower mineralization rate and slower fracture wound healing.

These data suggest that the miR-19b agomir accelerates fracture healing, that oe-WWP1 and oe-Smurf2 delay fracture healing, and that the miR-19b agomir + oe-WWP1 and miR-19b agomir + oe-Smurf2 treatments can reverse the delay in healing induced by oe-WWP1 and oe-Smurf2. miR-19b activates the KLF5/β-catenin signaling pathway through inhibition of WWP1/Smurf2, accelerating fracture healing in mice.

### BMSC-exos accelerate fracture healing in vivo by delivering miR-19b

In this study, exosomes derived from BMSCs (BMSC-exos) were characterized. TEM observation showed that BMSC-exos exhibited a cup-like or spherical morphology (Supplementary Fig. S2a), and the number of exosomes increased after induction. Dynamic light scattering (DLS) analysis data suggested that the diameter of MSC-exos was mainly between 30-100 nm (Supplementary Fig. S2b). Western blot detection of exosome surface markers also revealed that the expression levels of exosome markers (CD9, CD81, TSG101, HSP70, ALIX and Flotillin-1) in MSCs were increased (Supplementary Fig. S2c). We also detected the expression of miR-19b in BMSC-exos, and the data suggested that the expression of miR-19b was higher than that in MSCs (Supplementary Fig. S2d).

**Fig. 6:**
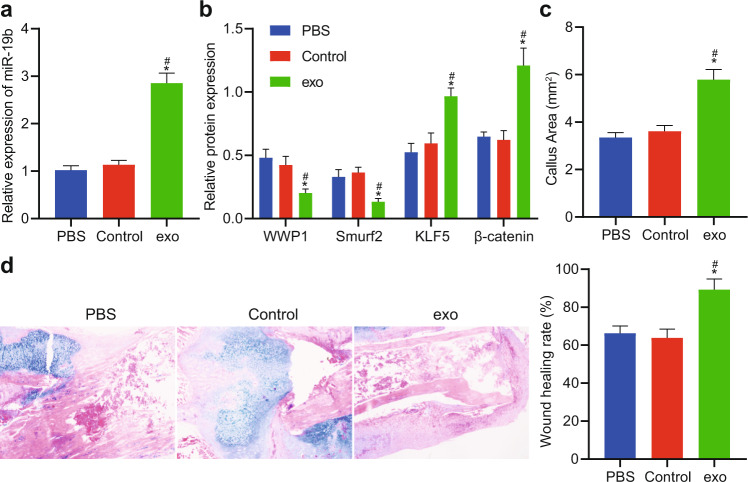
BMSC-exos promote fracture healing in mice. **a** PCR was used to detect the level of miR-19b. **b** Western blot analysis of the protein levels of WWP1, Smurf2, KLF5, and β-catenin in the calli of each group at week 0 after fracture, with β-actin as the internal reference protein. *p < 0.05 compared with the PBS group; ^#^*p* < 0.05 compared with the control group; NS *p* > 0.05. **c** Quantitative analysis of the callus area during femoral fracture healing at the 4th week after fracture. **d** HE/Alcian blue staining results for evaluating the mineralized callus area. The experimental results are measurement data and are expressed as the mean ± standard deviation values. For multiple comparisons, one-way ANOVA followed by Tukey’s post hoc test was performed. *n* = 5 in the weeks 0 and 4 after fracture.

On the 7th day after animal model establishment, calli were harvested from mice to quantify the expression of miR-19b. The miR-19b expression level in the exo group was higher than that in the PBS and control groups (Fig. 6a). In addition, the protein expression levels of WWP1, Smurf2, KLF5 and β-catenin were measured in mice of each group (Fig. 6b). The data suggested that WWP1 and Smurf2 were downregulated and KLF5 and β-catenin were upregulated in the exo group relative to the control group. These data suggest that injection of BMSC-exos can promote the expression of KLF5/β-catenin by inhibiting WWP1/Smurf2. At week 4 after the establishment of the fracture model, the healing area of the inner bone formation during femoral fracture healing in mice was evaluated by X-ray imaging (Fig. 6c). Statistical analysis suggested that the healing area was larger in the exo group than in the control group at the 4th week, and the remodeling process was observed. At the 4th week after fracture in mice, the fracture calli from the treated mice in each group were decalcified and stained with HE/Alcian blue. As shown in Fig. 6d, at the 4th week, the exo group had a higher mineralization proportion and better recovery ability of fracture wounds than the control group. The above data suggested that BMSC-exos upregulated miR-19b expression and accelerated fracture healing in mice.

## Discussion

Bones have repair potential following fractures, and the healing process is involved in bone repair and the restoration of structural integrity^19^. Healing of bone fractures restores the preinjury structure and function of the damaged skeleton, but nearly 10% of fractures cannot be repaired normally^1^. BMSCs possess multilineage differentiation capabilities, including osteogenesis and chondrogenesis^20^, which enable them to function as mediators to facilitate fracture repair^21^. The current study explored the role of exosomal miR-19b derived from BMSCs in fracture healing.

The experimental data revealed that miR-19b promoted the differentiation of human BMSCs into osteoblasts via targeting WWP1 and Smurf2. Notable evidence was obtained demonstrating that miRNAs play a pivotal role in bone homeostasis by orchestrating the expression of key genes related to osteoblasts and osteoclasts^22^. Other studies have documented that the fine-tuning effects of miRNAs in bone remodeling and fracture repair are achieved by controlling gene expression in bone cells^23^. miR-19b is one of the components included in exosomes derived from MSCs^24^ and can promote fracture healing during osteogenic differentiation^25^. Thus, we modulated miR-19b expression in BMSCs and in a mouse model to explore the resulting effects on osteogenic factors, bone cell mineralization and the healing status of modeled fractures. The observations suggested that miR-19b overexpression can facilitate the expression of osteogenic factors (Runx2, ALP, and Collagen I), bone cell mineralization and healing processes in mice. Consistent results were reported in a prior study, which emphasized that osteogenic differentiation was induced by ectopic miR-19b-3p expression, corresponding to elevated osteocalcin, osteopontin, and Runx2 levels^9^.

Furthermore, the targeting relationship between miR-19b and WWP1/Smurf2 was determined by an initial bioinformatic prediction followed by a confirmatory dual-luciferase reporter assay. miRNAs have the capacity to orchestrate gene expression by binding to complementary sites in mRNAs and reducing the stability and translation of the target mRNAs^26^. Ubiquitin E3 ligase-dependent protein degradation facilitates proteasomal degradation of important regulators of osteogenic functions. WWP1 accelerates the degradation of the osteogenic factor Runx2 to repress its osteogenic function, and decreasing the WWP1 level in BMSCs may constitute a promising strategy to suppress the impairment of osteoblastic function^27^. The ubiquitin E3 ligase Smurf2 was also found to orchestrate osteoblast function by augmenting proteasomal degradation of osteogenic proteins^28^. These observations thus further corroborate our findings that overexpression of WWP1 or Smurf2 led to degradation of their target protein KLF5 in BMSCs through ubiquitination to inhibit fracture healing. WWP1 and Smurf2 display similar inhibitory effects on KLF5^29^. For example, both use a binding domain and a catalytic mechanism to enhance the degradation of KLF5, and the degradation of KLF5 by both proteins is highly inhibited by the proteasome inhibitor MG132. The KLF5 protein is clearly controlled by the ubiquitin-proteasome pathway, and WWP1 negatively regulates the function of KLF5 in gene regulation by controlling its ubiquitination and degradation^30^. Consistent with these observations, another study documented that Smurf2 specifically destabilizes KLF5, that the degradation of KLF5 by Smurf2 is disrupted by the proteasome inhibitor MG132, and that Smurf2 ubiquitinates KLF5^11^. Of note, KLF5 was reported to mediate the proliferative potential and osteogenic differentiation of human periodontal ligament cells by restricting ALP activity and Runx2 expression^31^. Our mechanistic studies further demonstrated that KLF5 knockdown delayed fracture healing by modulating the Wnt/β-catenin signaling pathway. The activation of β-catenin can increase bone formation and decrease bone resorption, resulting in accelerated bone fracture healing, and thus, Wnt/β-catenin signaling activation should be sustained during fracture healing to intensify repair^32^. In a previous mouse model of closed tibial fracture, inactivation of β-catenin in chondrocytes impeded fracture healing due to restricted chondrogenesis and endochondral ossification^33^. Our in vivo experiments in mouse models also substantiated that BMSC-exos containing increased levels of miR-19b enhanced fracture healing *via* the KLF5/β-catenin signaling pathway by targeting WWP1 or Smurf2.

Thus, we provided evidence confirming that upregulation of miR-19b and activation of KLF5/β-catenin signaling may potentially be clinically viable targets in the treatment of fractures. More importantly, mesenchymal stem cell-derived exosomal miR-19b represses the expression of WWP1 or Smurf2 and elevates KLF5 expression through the Wnt/β-catenin signaling pathway, thereby facilitating fracture healing. As we further elucidate the epigenetic regulatory mechanisms underlying fracture healing, there is great potential for translational application of mesenchymal stem cell-derived exosomal miR-19b.

## Supplementary information

supplementary information

## Data Availability

The datasets generated and/or analyzed during the current study are available from the corresponding author on reasonable request.
