# Supplementary material for: miR-19b enhances osteogenic differentiation of mesenchymal stem cells and promotes fracture healing through the WWP1/Smurf2-mediated KLF5/β-catenin signaling pathway
Source: Exp Mol Med. 2021 May 25;53(5):973–85. doi: 10.1038/s12276-021-00631-w (PMC8178348; doi:10.1038/s12276-021-00631-w)
Supplement: Supplementary file 1 — supplementary information [file 12276_2021_631_MOESM1_ESM.pdf]

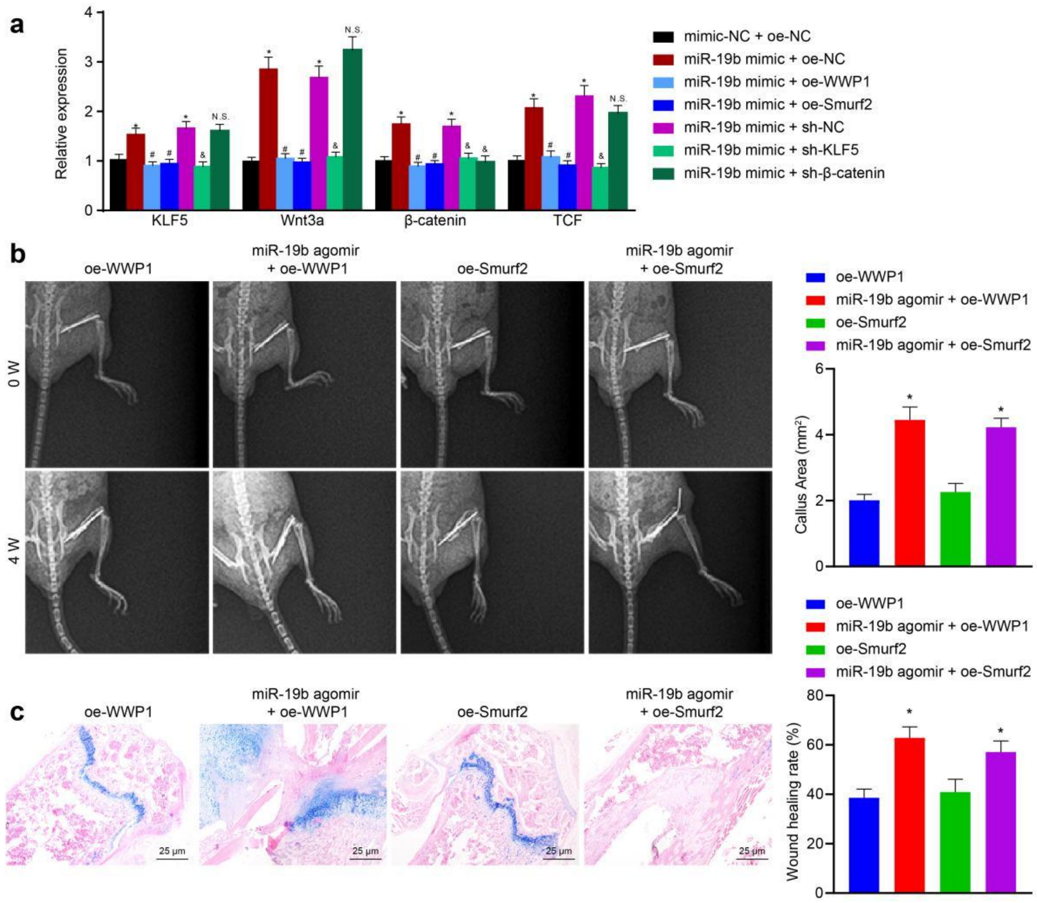

Fig. S1 miR-19b promotes fracture healing in mice through the WWP1/Smurf2 axis. a, The expression of KLF5 and β-catenin was measured by qPCR. * $p < 0.05$ versus the mimic-NC + sh-NC group, # $p < 0.05$ versus the miR-19b mimic + oe-NC group, & $p < 0.05$ versus the miR-19b mimic + sh-NC group, NS $p > 0.05$. b, Representative X-ray image capture for femoral healing and changes over time at 0th and 4th week after fracture. c, At 4th week after femoral fracture, callus sections underwent staining with HE/Alcian blue, and the histology of fracture callus was observed. Blue indicates positive cartilage, and pink indicates mineralized callus area. The experimental results are measurement data and expressed as mean ± standard deviation. For multiple comparisons, one-way ANOVA followed by Tukey post hoc test was performed. n = 5 in the 0th and 4th week after fracture.

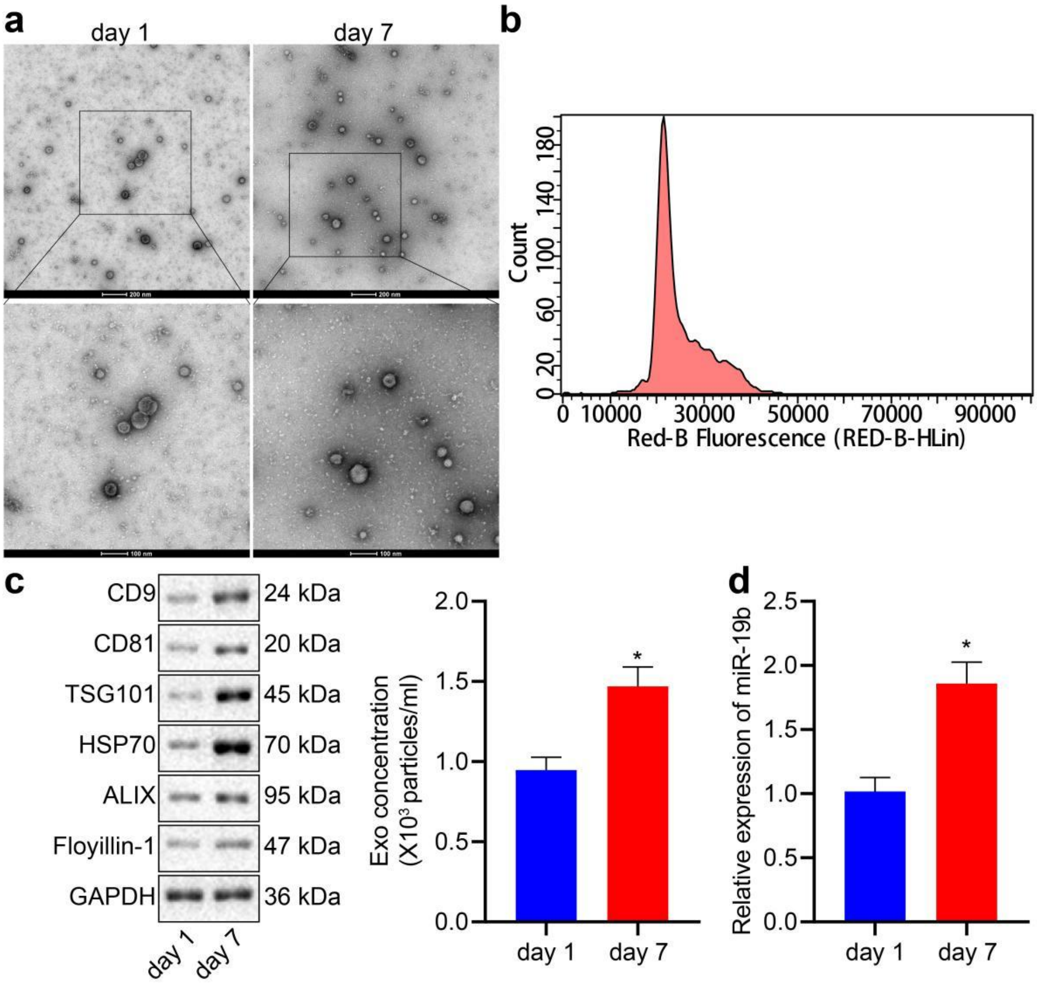

Fig. S2 Identification of BMSCs-exo and quantitation of miR-19b in BMSCs-exo. a, Morphology of exosomes observed by TEM. b, Size distribution of exosomes measured by DLS. c, Exosome surface specific marker proteins detected by Western blot analysis. d, miR-19b expression in exosomes detected by RT-qPCR. The experimental results are measurement data and expressed as mean ± standard deviation. For comparisons between two groups, independent sample t test was conducted. * $p < 0.05$ versus day 1. The experiment was repeated 3 times.
